# Retrospective Quantitative Genetic Analysis and Genomic Prediction of Global Wheat Yields

**DOI:** 10.3389/fpls.2020.580136

**Published:** 2020-08-27

**Authors:** Philomin Juliana, Ravi Prakash Singh, Hans-Joachim Braun, Julio Huerta-Espino, Leonardo Crespo-Herrera, Thomas Payne, Jesse Poland, Sandesh Shrestha, Uttam Kumar, Arun Kumar Joshi, Muhammad Imtiaz, Mohammad Mokhlesur Rahman, Fernando Henrique Toledo

**Affiliations:** ^1^International Maize And Wheat Improvement Center (CIMMYT), Texcoco, Mexico; ^2^Campo Experimental Valle de Mexico, Instituto Nacional de Investigaciones Forestales, Agricolas y Pecuarias (INIFAP), Chapingo, Mexico; ^3^Wheat Genetics Resource Center, Department of Plant Pathology, Kansas State University, Manhattan, KS, United States; ^4^CIMMYT, New Delhi, India; ^5^Borlaug Institute for South Asia (BISA), New Delhi, India; ^6^CIMMYT, Islamabad, Pakistan; ^7^Regional Agricultural Research Station, Bangladesh Agricultural Research Institute (BARI), Jamalpur, Bangladesh

**Keywords:** wheat, grain yield, quantitative genetics, genomic prediction, genotype x environment

## Abstract

Breeding for grain yield (GY) in bread wheat at the International Maize and Wheat Improvement Center (CIMMYT) involves three-stage testing at Obregon, Mexico in different selection environments (SEs). To understand the efficiency of selection in the SEs, we performed a large retrospective quantitative genetics study using CIMMYT’s yield trials evaluated in the SEs (2013–2014 to 2017–2018), the South Asia Bread Wheat Genomic Prediction Yield Trials (SABWGPYTs) evaluated in India, Pakistan, and Bangladesh (2014–2015 to 2017–2018), and the Elite Spring Wheat Yield Trials (ESWYTs) evaluated in several sites globally (2003–2004 to 2016–2017). First, we compared the narrow-sense heritabilities in the Obregon SEs and target sites and observed that the mean heritability in the SEs was 44.2 and 92.3% higher than the mean heritabilities in the SABWGPYT and ESWYT sites, respectively. Second, we observed significant genetic correlations between a SE in Obregon and all the five SABWGPYT sites and 65.1% of the ESWYT sites. Third, we observed high ratios of response to indirect selection in the SEs of Obregon with a mean of 0.80 ± 0.21 and 2.6 ± 5.4 in the SABWGPYT and ESWYT sites, respectively. Furthermore, our results also indicated that for all the SABWGPYT sites and 82% of the ESWYT sites, a response greater than 0.5 can be achieved by indirect selection for GY in Obregon. We also performed genomic prediction for GY in the target sites using the performance of the same lines in the SEs of Obregon and observed moderate mean prediction accuracies of 0.24 ± 0.08 and 0.28 ± 0.08 in the SABWGPYT and ESWYT sites, respectively using the genotype x environment (GxE) model. However, we observed similar accuracies using the baseline model with environment and line effects and no advantage of modeling GxE interactions. Overall, this study provides important insights into the suitability of the Obregon SEs in breeding for GY, while the variable genomic predictabilities of GY and the high year-to-year GY fluctuations reported, highlight the importance of multi-environment testing across time and space to stave off GxE induced uncertainties in varietal yields.

## Introduction

Increasing the grain yield (GY) potential of bread wheat (*Triticum aestivum* L.) and developing resilient varieties are critical to ensure food security amidst the low (0.9%) global average rate of increase (Ray et al., 2013) and escalating challenges like fluctuating temperatures, low precipitation, erratic rainfall patterns, and extreme weather conditions (Wheeler and von Braun, 2013; Curtis and Halford, 2014; Trnka et al., 2014; Tack et al., 2015; Zampieri et al., 2017; Hatfield and Dold, 2018). However, the identification of high-yielding widely adapted varieties is constrained by the interactions of genotypes with the environments (GxE interactions) that result in genotype rank changes across environments and reduce the response to selection (Haldane, 1946; Allard and Bradshaw, 1964; Eberhart and Russell, 1966; Knight, 1970). To stave off GxE induced uncertainties in varietal yields and the impending risks of crop failure, the International Maize and Wheat Improvement Center (CIMMYT) performs intensive three-stage GY testing at its primary yield testing site, the Norman E. Borlaug Experimental Research Station, Ciudad Obregon, Sonora, Mexico (27°29′N, 109°56′W) and also analyzes GY data returned by collaborating national partners from its target population of environments (TPEs) to identify lines with temporal and spatial stability over a range of environmental conditions (Crossa et al., 1991; Fox, 1994; Cooper and Byth, 1996; Cooper et al., 1997; Braun et al., 2010). These strategies adopted by CIMMYT have bolstered the development of high-yielding, widely adapted stable wheat lines (Rajaram and Skovmand, 1977; Braun et al., 1996; Singh and Trethowan, 2007; Lage et al., 2008) that have a remarkable impact globally, especially in developing countries (Byerlee and Moya, 1990; Heisey et al., 2002; Lantican et al., 2005; Lantican et al., 2016).

The GY selection-environments (SEs) in Obregon are well tailored to represent different planting systems, irrigation systems and abiotic stresses in the spring bread wheat target mega-environments (MEs) (Rajaram et al., 1993; Braun et al., 2010). The first year/stage (Stage 1) of GY testing in Obregon involves the evaluation of about 9,000 lines that were selected from the head-rows in the fully irrigated bed planting environment (Stage 1 irrigated BP). The second year/stage (Stage 2) involves the evaluation of 1,092 lines (~12% of 9,000 lines) selected from Stage 1 in six environments including the fully irrigated bed planting (Stage 2 irrigated BP), fully irrigated flat planting (Stage 2 irrigated FP), reduced irrigation (Stage 2 reduced irrigation), drought-stressed (Stage 2 drought), early-sown heat stressed (Stage 2 early heat), and late-sown heat stressed (Stage 2 late heat) environments. The third year/stage (Stage 3) of testing involves 280 lines (~25% of 1,092 lines) selected from Stage 2 that are evaluated in three environments including the fully irrigated bed planting (Stage 3 irrigated BP), drought-stressed (Stage 3 drought), and late-sown heat stressed (Stage 3 late heat) environments. In parallel with the Stage 3 of GY testing in Obregon, the first GY testing at CIMMYT’s major TPEs in South Asia is done through the South Asia Bread Wheat Genomic Prediction Yield Trial (SABWGPYT). This trial initiated in 2014 comprises 540 lines (~50% of 1,092 lines) selected for high GY from Stage 2 of yield testing in Obregon, that are evaluated in fully irrigated flat seed beds at the following main wheat growing regions of South Asia: (i) Pakistan, Faisalabad (31°25’N, 73°4’E) (ii) Bangladesh, Jamalpur (24°55′N, 89°57′E) (iii) Research stations of the Borlaug Institute for South Asia in India, including Ludhiana, Punjab (30°54′N, 75°51′E, representing the North-Western Plain Zone), Pusa, Bihar (25°59′N, 85°41′E, representing the North-Eastern Plain Zone), and Jabalpur, Madhya Pradesh (23°10’N, 79°55’E, representing the Central Zone).

The lines with high and stable GY relative to checks in the fully irrigated trials at the South Asia TPEs and in the Obregon SEs that also possess good to moderate drought and heat tolerance are selected and comprise the Elite Spring Wheat Yield Trials (ESWYTs). This includes 50 lines distributed globally on request every year that are targeted to the irrigated wheat growing environments with mostly favorable temperatures during the main crop season, including the Northwestern Gangetic Plains of South Asia, Egypt, Northwestern Mexico (Obregon), various spring wheat-growing areas of Turkey, Afghanistan, Iran, etc. In addition to the ESWYTs, three other international wheat yield trials including the semi-arid wheat yield trials, high-temperature wheat yield trials and the high rainfall wheat yield trials targeted to the drought-stressed, heat-stressed, and high rainfall regions, respectively are also distributed annually by CIMMYT (Trethowan and Crossa, 2007). While CIMMYT’s international yield trials have proved to be extremely useful for characterizing relationships between selection and target sites, genotype adaptations and GxE interactions (Krull et al., 1968; Byth et al., 1976; Peterson and Pfeiffer, 1989; DeLacy et al., 1993; Trethowan et al., 2001; Lillemo et al., 2004), an extensive retrospective quantitative genetics study focusing on understanding the GY heritabilities and genetic correlations (GCs) and their effect on the response to selection for GY is lacking.

Heritability is a key parameter in quantitative genetics that is important to plant breeders as it expresses the correspondence between GY phenotypic and breeding values and determines the response to selection (Fisher, 1918; Falconer, 1960; Piepho and Möhring, 2007). While the broad-sense heritability represents the phenotypic variance that can be attributed to both additive and non-additive genetic variance, the narrow-sense heritability represents the proportion of phenotypic variance that can be attributed to the additive genetic variance (Jacquard, 1983; Nyquist, 1991; Kruijer et al., 2015). Two other critical quantitative genetics parameters that are pivotal in understanding the efficiency of selecting in a few SEs for a wide range of TPEs include: (i) the genetic correlations (GCs) which determine the extent to which GY in two environments is influenced by the same genes and (ii) the ratio of correlated response to indirect selection in the SEs relative to direct selection in the TPEs (Falconer, 1960; DeLacy et al., 1996). In addition to understanding the quantitative genetic parameters associated with GY, it is also essential to evaluate genomic approaches that can minimize the adverse effects of GxE interactions on the response to selection for GY. In this regard, genomic selection (GS) where the genomic-estimated breeding values (GEBVs) of lines obtained from genome-wide markers are used in selecting individuals (Meuwissen et al., 2001) has been found to be promising for improving GY in wheat (Zhao et al., 2013; Charmet et al., 2014; Juliana et al., 2018; Lozada et al., 2019). Since it has the potential to increase the selection accuracy and reduce the costs associated with phenotyping (Heffner et al., 2009; Crossa et al., 2014; Voss-Fels et al., 2018), it can be very beneficial in effectively selecting lines for the TPEs.

Recognizing the importance of quantitative genetic parameters in understanding the efficiency of CIMMYT’s yield testing strategies, we designed this study with the following key objectives: (i) estimate the GY narrow-sense heritabilities using 36 trials evaluated in the SEs of Obregon and 534 trials evaluated in the TPEs, (ii) estimate the GCs and the rates of response to selection using four cohorts of breeding lines evaluated in the SEs of Obregon and in the SABWGPYTs and two cohorts evaluated in the SEs of Obregon, SABWGPYTs, and ESWYTs, (iii) cluster the sites in the TPEs based on their GCs to identify sites where the lines have similar patterns of GY performance to facilitate better targeting of lines for those TPEs. In addition, to evaluate the prospects of implementing GS for targeting lines to the TPEs, we first determined the genomic prediction accuracies (PAs) for GY in the target sites using the GY of the same lines in the SEs in Obregon for 1,424 selection and target environment pairs. For this we used a GxE model with genomic effects, environment effects, and genotype x environment effects that has been shown to boost the PAs (Burgueño et al., 2012; Heslot et al., 2013; Crossa et al., 2016; Jarquín et al., 2017; Pérez-Rodríguez et al., 2017), and compared the PAs to those from a baseline model with only the main effects of the environment and the lines (EL model) to understand the advantage of modeling GxE interactions. Moreover, we also compared the PAs from the GxE model with the phenotypic correlations between the environments to comprehend the relationships between them. Furthermore, we also partitioned the phenotypic GY variance between the selection and target environments into genetic, environment, genotype x environment, and error variance to decipher the relative contribution of these components towards GY.

## Materials and Methods

### Populations, Phenotyping, and Best Linear Unbiased Estimates for Grain Yield

The yield trial lines used in this study were developed by the CIMMYT wheat breeding program using the selected bulk breeding approach, which involves early generation visual selection for phenological traits, agronomic type, rust resistance, tillering capacity, spike fertility, grain size, and overall grain health. GY in all the trials was measured in tonnes/hectare obtained from the plot-based harvested grain weight. In the Stage 1 of GY testing in Obregon, 6,408, 7,987, and 8,182 lines were evaluated in the irrigated BP environment in the 2013–2014, 2014–2015, and 2015–2016 cycles, respectively. The lines were sown on raised beds during the optimum planting time from the third week of November to the first week of December and they received an optimum irrigation of about 500 mm of water in five irrigations throughout the cycle. The trials were laid out in an alpha-lattice design with two replications and two checks.

In the Stage 2 of yield testing, 1,092 lines were evaluated each year during the 2013–2014 to 2016–2017 cycles in all the six environments, except in the 2015–2016 cycle where the lines were not evaluated in the Stage 2 early heat and Stage 2 late heat environments. The lines evaluated in the Stage 2 irrigated BP and Stage 2 irrigated FP environments were planted in raised and flat seed beds, respectively, during the optimum planting time and received a total of about 500 mm of water in five irrigations. The lines evaluated for drought-stress in the Stage 2 reduced irrigation and Stage 2 drought environments were planted in raised and flat seed beds respectively, during the optimum planting time. While the Stage 2 reduced irrigation environment received a total of about 250 mm of water in two irrigations, the Stage 2 drought environment received a total of 180 mm of water through drip irrigation. Evaluation of the lines for GY under high temperatures during the juvenile growth stage (Stage 2 early heat) and during the heading and grain-filling stages (Stage 2 late heat) were achieved by sowing the lines early in mid-October (30 days before he optimum planting time) and late in the last week of February (90 days after the optimum planting time), respectively. Both the heat-stressed environments received a total of 500 mm of water in five irrigations. In the Stage 3 of yield testing, 280 lines were evaluated each year during the 2014–2015 to 2017–2018 cycles in the irrigated BP, drought, and late heat environments, as described above.

The 540 lines in the SABWGPYTs were grown in an alpha-lattice design with two replications during the 2014–2015 to 2017–2018 cycles. While the lines were evaluated in India Jabalpur, India Ludhiana, and India Pusa during all the cycles, they were evaluated in Bangladesh Jamalpur during the 2016–2017 cycle only and in Pakistan Faisalabad during the 2015–2016 and 2016–2017 cycles only. The 24^th^ to the 37^th^ ESWYTs comprising 700 lines with 50 lines in each ESWYT (46 lines, three CIMMYT checks, and one local check) were evaluated in two replications in several sites during the 2003–2004 (24^the^ ESWYT) to 2016–2017 (37^th^ ESWYT) cycles. However, all the ESWYTs were not evaluated in all the sites due to several factors like the lack of capacity for GY testing, the importance for bread wheat in the site etc. (Lage et al., 2008) and hence only the 60 sites that had been evaluated at least five of the 14 ESWYTs were included in the analysis. This included sites in 17 countries including Afghanistan, Algeria, Argentina, Bangladesh, Canada, Chile, Egypt, India, Iran, Mexico, Nepal, Pakistan, Portugal, South Africa, Spain, Sudan, and Turkey that are described in **Supplementary Table 1**.

To obtain the best linear unbiased estimates for GY in all the datasets, we used the below mixed model in the ASREML ‘R’ package (Gilmour, 1997):

(1) $$y_{ijkl}=\mu +g_{i}+t_{j}+r_{k(j)}+b_{l(jk)}+\epsilon_{ijkl}\;$$

where *y_ijkl_* represents the unadjusted GY, μ represents the overall mean, *g_i_* represents the fixed effect of the genotype, *t_j_* represents the random effect of the trial assumed to be independent and identically distributed (IID) $\left(t_{j}\sim N\;\left(0,\;\sigma_{t}^{2}\right)\right)$, *r_k_*_(_*_j_*_)_ represents the random effect of the replicate within the trial assumed to be IID $\left(r_{k\left(j\right)}\sim N\;\left(0,\;\sigma_{r}^{2}\right)\right)$, *b_l_*_(_*_jk_*_)_ represents the random effect of the incomplete block within the trial and the replicate assumed to be IID $\left(b_{m\left(jk\right)}\sim N\;\left(0,\;\sigma_{b}^{2}\right)\right)$ and *ϵ_ijkl_* is the residual assumed to be IID $\left(\epsilon_{ijkl}\sim \;N\;\left(0,\;\sigma_{\epsilon }^{2}\right)\right)$. The GY BLUEs for all the lines in the Stage 1, Stage 2, Stage 3 of yield testing, the SABWGPYTs and the ESWYT sites are given in **Supplementary Tables 2** and **3**.

### Genotyping

Genome-wide markers for all the lines used in this study were obtained using the genotyping-by-sequencing (GBS) method (Poland et al., 2012). The marker polymorphisms were called using the TASSEL (Trait Analysis by aSSociation Evolution and Linkage) version 5 GBS pipeline (Bradbury et al., 2007; Glaubitz et al., 2014). We then performed single nucleotide polymorphism discovery by filtering for a minor allele frequency of 0.01, followed by aligning the resulting 6,075,743 GBS tags to the reference genome (RefSeq v1.0) of bread wheat (IWGSC, 2018). Further filtering of the tags as described in Juliana et al. (2019) resulted in 78,662 markers that passed at least one of the filters. We then filtered the markers with greater than 60% missing data, minor allele frequency lesser than 5% and heterozygosity less than 10%, and also the lines that had greater than 50% missing data. This resulted in the following number of lines and markers for the different yield trials in Obregon and the SABWGPYTs: (i) Stage 1 2012–2013—947 lines and 6,071 markers; (ii) Stage 1 2013–2014—6,408 lines and 8,416 markers; (iii) Stage 1 2014–2015—7,987 lines and 11,982 markers; (iv) Stage 1 2015–2016—8,182 lines and 11,518 markers; (v) Stage 2 2013–2014—947 lines and 6,071 markers; (vi) Stage 2 2014–2015—1,012 lines and 5,963 markers; (vii) Stage 2 2015–2016—1,052 lines and 8,402 markers; (viii) Stage 2 2016–2017—1,040 lines and 8,312 markers; (ix) Stage 3 2014–2015—269 lines and 6,180 markers; (x) Stage 3 2015–2016—263 lines and 5,399 markers; (xi) Stage 3 2016–2017—272 lines and 6,356 markers; (xii) Stage 3 2017–2018—264 lines and 5,768 markers; (xiii) SABWGPYT 2014–2015—508 lines and 5,600 markers; (xiv) SABWGPYT 2015–2016—487 lines and 6,122 markers; (xv) SABWGPYT 2016–2017—515 lines and 7,752 markers; and (xvi) SABWGPYT 2017–2018—505 lines and 7,474 markers. Similarly, in the 14 ESWYTS, the number of lines and markers after filtering ranged from 36 to 43 and from 7,742 to 12,441, respectively. Marker missing data was imputed using the linkage-disequilibrium based k-nearest neighbor imputation method in TASSEL (Money et al., 2015) and the unfiltered genotyping data is available in https://doi.org/10.6084/m9.figshare.12609368.v2.

### Statistical Analysis of the Grain Yield Data in the Target Sites and Narrow-Sense Heritabilities in the Selection and Target Environments

To understand the GY performance of lines in the SABWGPYTs (2014–2014 to 2017–2018) and in the ESWYTs (2003–2004 to 2016–2017), we performed statistical analysis of the GY BLUEs within each yield trial and calculated the mean, median, range, standard deviation, standard error of the mean, and variance. We then estimated the line mean narrow-sense heritabilities for GY across replications in the SEs and in the target sites using the formula:

(2) $$h^{2}=\;\frac{\sigma_{g}^{2}}{\sigma_{g}^{2}+\;\frac{\sigma_{\epsilon }^{2}}{nreps}}$$

where $\sigma_{g}^{2}$ represents the genetic variance, $\sigma_{\epsilon }^{2}$ represents the error variance, and *nreps* represents the number of replications. The genetic and error variances in each trial were estimated using the average information-restricted maximum likelihood algorithm (Gilmour et al., 1995) in the ‘R’ package ‘heritability’ (Kruijer et al., 2015). The datasets that were used for the estimation of heritabilities include: (i) 23,524 lines evaluated in the SEs of Obregon between 2013–2014 and 2017–2018 in 36 trials; (ii) 2,015 lines evaluated in the five SABWGPYT sites between 2014–2015 and 2017–2018 in 15 trials; and (iii) 700 lines evaluated in 60 ESWYT sites between 2003–2004 and 2016–2017 in 519 trials.

### Genetic Correlations and Clustering of Target Sites Based on Genetic Correlations

We estimated GCs from the genetic covariances calculated in the ‘R’ package EMMREML (Akdemir and Okeke, 2015) using the formula (Falconer, 1960),

(3) $$r_{A}=\frac{COV_{XY}}{\sqrt{var_{X}var_{Y}}}$$

where *r_A_* represents the genetic correlation of GY in two environments, *cov_XY_* represents the covariance for GY in the two environments, and *var_X_* and *var_Y_* are the variances for GY in the two environments. The function ‘emmreml Multivariate’ in EMMREML accounts for the additive genetic (co)variance matrix of GY in different environments, that is calculated using markers and solves a multivariate Gaussian mixed model with a known covariance structure.

The GCs between GY evaluated in the Stage 1 and Stage 2 SEs and the target SABWGPYTs were evaluated in four sets of breeding lines or cohorts, as follows: (i) **Cohort 1**: 508 lines in Stage 1 2012–2013, Stage 2 2013–2014, and SABWGPYT 2014–2015; (ii) **Cohort 2**: 487 lines in Stage 1 2013–2014, Stage 2 2014–2015, and SABWGPYT 2015–2016; (iii) **Cohort 3**: 515 lines in Stage 1 2014–2015, Stage 2 2015–2016, and SABWGPYT 2016–2017; and (iv) **Cohort 4**: 505 lines in Stage 1 2015–2016, Stage 2 2016–2017, and SABWGPYT 2017–2018. Similarly, the GCs between GY evaluated in all the SEs of Obregon, and in the target SABWGPYT and ESWYT sites were obtained in two cohorts as follows: (i) **Cohort 1**: 42 lines in Stage 1 2012–2013, Stage 2 2013–2014, Stage 3 2014–2015, SABWGPYT 2014–2015, and ESWYT 2015–2016; (ii) **Cohort 2**: 43 lines in Stage 1 2013–2014, Stage 2 2014–2015, Stage 3 2015–2016, SABWGPYT 2015–2016, and ESWYT 2016–2017. In addition, we also obtained the GCs between the SABWGPYT sites across the years, the 69 sites in the ESWYT 2015–2016, and the 71 sites in the ESWYT 2016–2017. The significance of all the GCs were tested and the p-values for the test of significance were obtained. We then used the GCs between the 44 ESWYT sites that had evaluated both the 36^th^ and 37^th^ ESWYTs to cluster the sites using the hierarchical clustering approach and the dendrogram was cut into five branches and visualized using the ‘R’ package, ‘pheatmap’ (Kolde, 2012). The clustering patterns of the ESWYT sites in the two different years were then analyzed to identify consistent patterns.

### Rate of Response to Selection

To understand the effectiveness of indirect selection in the SEs of Obregon relative to that of direct selection in the TPEs, we calculated the ratio of response to indirect vs direct selection using the formula (Falconer, 1960):

(4) $$\frac{CR_{X}}{R_{X}}=r_{A}\frac{i_{Y}}{i_{X}}\frac{h_{Y}}{h_{X}}$$

where *CR_X_* is the correlated response to GY selection in the target environment (X) resulting from selection applied to GY in the SE, *R_X_* is the direct response to GY selection in the target environment, *r_A_* is the GC between the selection and target-environments, *i_Y_* and *i_X_* are the intensities of selection in the selection and target-environments, respectively (which we assumed to be the same), and *h_Y_* and *h_X_* are the narrow-sense heritabilities for GY in the selection and target-environments, respectively.

### Genomic Prediction for Grain Yield in the Target Sites Using Their Yields in the Selection-Environments of Obregon

The ability of GY evaluations in the SEs of Obregon to predict GY in the target sites was assessed using four cohorts of lines in the SABWGPYTs and two cohorts of lines in the ESWYTs. To model the effects of the genotypes, environments and the GxE interactions in a GxE model, we used the reaction norm framework (Jarquín et al., 2014) and the model can be represented as:

(5) $$y=\mu 1+Z_{y}\beta_{y}+Z_{g}u_{1}+u_{2}+\epsilon$$

where ***y*** represents the vector of GY BLUEs; *μ* represents the general mean; ***Z****_y_* represents the incidence matrix for the environment; ***β****_y_* represents the random effect of the environment assumed to be multivariate normal $\beta_{y}\sim MN\left(0,\;\sigma_{y}^{2}I\right)$; ***Z****_g_* represents the incidence matrix connecting the lines with the GY BLUEs; ***u***_1_ represents the random effect of the lines; ***u***_2_ represents the GxE interaction assumed to be multivariate normal $u_{2}\sim MN\left(0,\sigma_{gy}^{2}\left(Z_{g}GZ_{g}^{'}\right)\#\left(Z_{y}Z_{y}^{'}\right)\right)$, where # denotes the Hadamard product (cell-by-cell) of the two matrices in parentheses (Jarquín et al., 2014), and **ϵ** represents the residuals assumed to be multivariate normal and distributed as $\epsilon \sim MN\left(0,\sigma_{\text{ϵ}}^{2}I\right)$. We also performed GY predictions using the EL model with environment and line effects and can be represented as:

(6) $$y=\mu 1+\;Z_{y}\beta_{y}+Z_{l}\beta_{l}+\;\epsilon \;$$

where ***y*** represents the vector of GY BLUEs, *μ* represents the general mean, ***Z****_y_* ***β****_y_* and **ϵ** represent the same as in Equation 5, ***Z****_l_* represents the incidence matrix for the lines and ***β****_l_* represents the random effect of the lines such that it is multivariate normal $\beta_{l}\sim MN\left(0,\;\sigma_{l}^{2}I\right)$, and $\sigma_{l}^{2}$ represents the variance of the lines. Both the GxE model and the EL model were fitted in the BGLR ‘R’ package and the Pearson’s correlations between the predicted values and the observed values were calculated as the PAs. To understand the relationships between PAs and the phenotypic correlations between the SEs ad TPEs, we also obtained the Pearson’s correlations between them and visualized them using the ‘R’ package ggplot2 (Wickham, 2009).

## Results

### Statistical Analysis of the Grain Yield Data and Grain Yield Progress

Statistical analysis of the GY data (**Supplementary Table 4**) indicated that in the SABWGPYT sites, the highest mean GY in all the years was observed in India Jabalpur where the mean GY across years ranged between 6.5 and 8.3 t/ha, followed by India Ludhiana (5.4 to 7 t/ha), India Pusa (4.4 to 6.1 t/ha), Pakistan Faisalabad (3.4 to 4.6 t/ha), and Bangladesh Jamalpur (3.1 t/ha). The mean GY from the 2014–2015 to the 2017–2018 cycle had increased by 27.3% in India Jabalpur, 31.2% in India Ludhiana, and 40.5% in India Pusa over the base mean GY in the 2014–2015 cycle. Similarly, the mean GY in Pakistan Faisalabad had increased by 36.8% across the two cycles (2015–2016 and 2016–2017).

In the ESWYTs, the mean GY across 14 years had increased by 24% from 4.6 ± 2.5 t/ha in the 24^th^ ESWYT to 5.7 ± 2 in the 37^th^ ESWYT. The highest mean yields in the most recent ESWYT analyzed were observed in Turkey Adana (11.1 t/ha), Egypt Sids (10 t/ha), Egypt Gemmeiza (8.06 t/ha), and Egypt Ety-El-Barud (7.9 t/ha). We also observed highly variable and non-linear GY trends across the years in several ESWYT sites (**Supplementary Figures 1** and **2**). However, a clear increasing trend in the mean GY (considering the mean GY in the first evaluated ESWYT and the highest mean GY of the two most recent ESWYTs) was observed in several sites including, (i) Afghanistan Darul Aman: 5.5 fold or 445.5% increase in 12 years, (ii) Canada Swift Current: 3.8 fold or 279% increase in 12 years, (iii) Pakistan Bahawalpur: 2.8 fold or 176.5% increase in 10 years, (iv) Pakistan Tandojam: 2.5 fold or 151.2% increase in 10 years, (v) Pakistan Islamabad: 2.1 fold or 111.8% increase in 13 years, and (vi) India Gurdaspur: 2.1 fold or 106% increase in 9 years.

**Figure 1:**
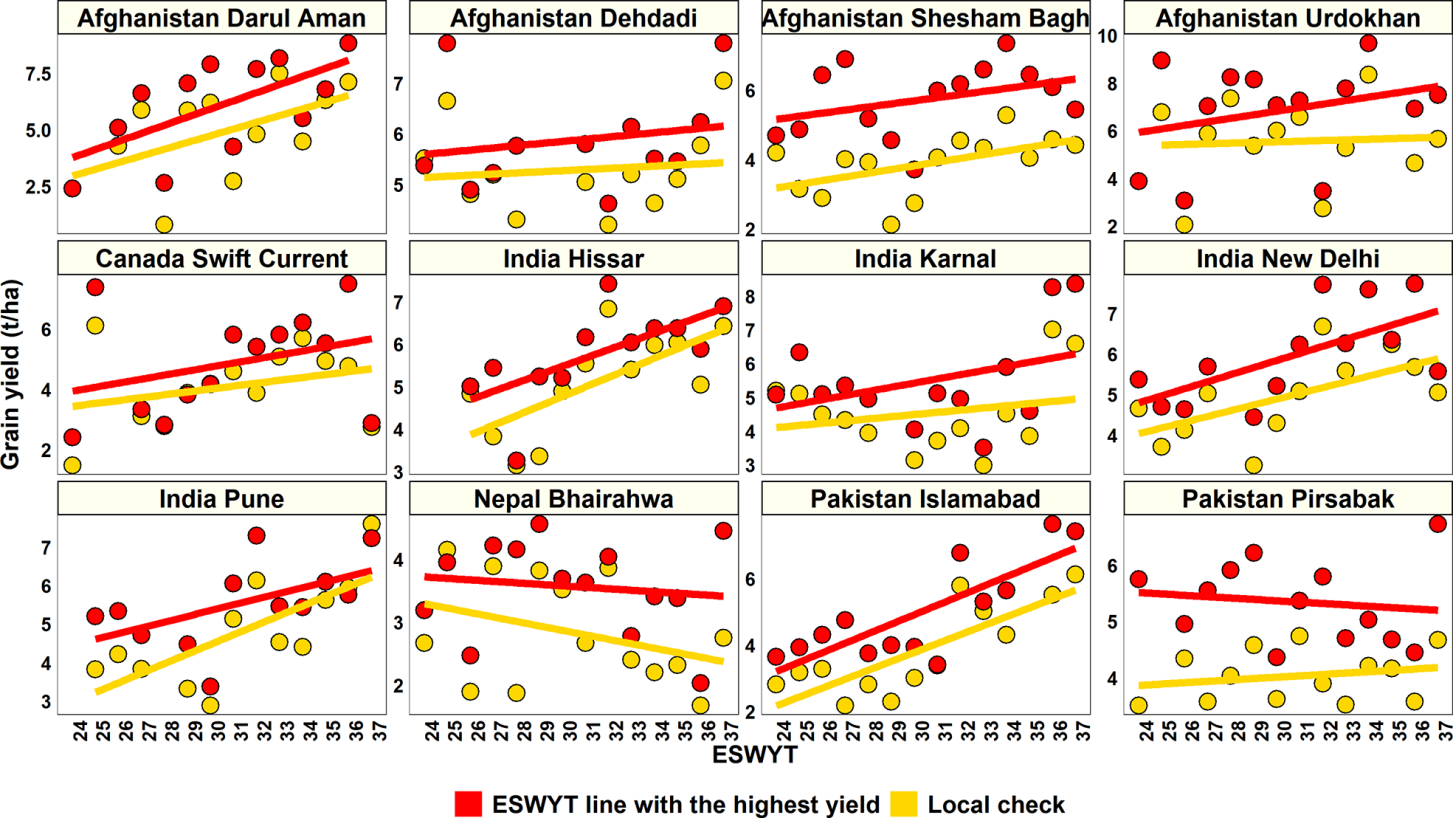
Relative grain yield performance of the highest yielding Elite Spring Wheat Yield Trial (ESWYT) line in each year over the grain yield of the local check in that year across 12 sites in the 24^th^ to the 37^th^ ESWYT.

The relative performance of the highest yielding ESWYT line in each year over the GY of the local check in that year was analyzed in 12 sites where the ESWYTs were evaluated in a higher number of years (12–14 years). We observed a clear superiority of the highest yielding ESWYT lines over the local check (**Figure 1**) in 144 out of the 152 (94.7%) site-year combinations. While the mean increase in GY of the highest yielding ESWYT line over the local check in the 144 site-year combinations was 1.1 ± 0.7 t/ha (29.5 ± 28.6% increase), it ranged from 0.03 to 3.54 t/ha (0.6 to 237.2% increase). In addition, we also observed a clear increasing trend in the GY of the highest yielding ESWYT line in most sites from the 24^th^ to the 37^th^ ESWYT.

### Narrow-Sense Heritabilities in the Selection and Target Environments

The highest mean narrow-sense heritabilities were observed in the SEs of Obregon (0.75 ± 0.11), followed by the SABWGPYT sites (0.52 ± 0.13) and the ESWYT sites (0.39 ± 0.29) (**Supplementary Table 5**). The mean narrow-sense heritabilities in the Obregon yield testing stages were: 0.65 ± 0.06 in Stage 1, 0.79 ± 0.1 in Stage 2, and 0.7 ± 0.12 in Stage 3 of GY testing. In the SABWGPYT sites, the highest mean narrow-sense heritabilities were observed in India Ludhiana (0.56 ± 0.08), followed by India Pusa (0.55 ± 0.14), India Jabalpur (0.53 ± 0.16), Pakistan Faisalabad (0.49 ± 0.15), and Bangladesh Jamalpur (0.32).

**Figure 2:**
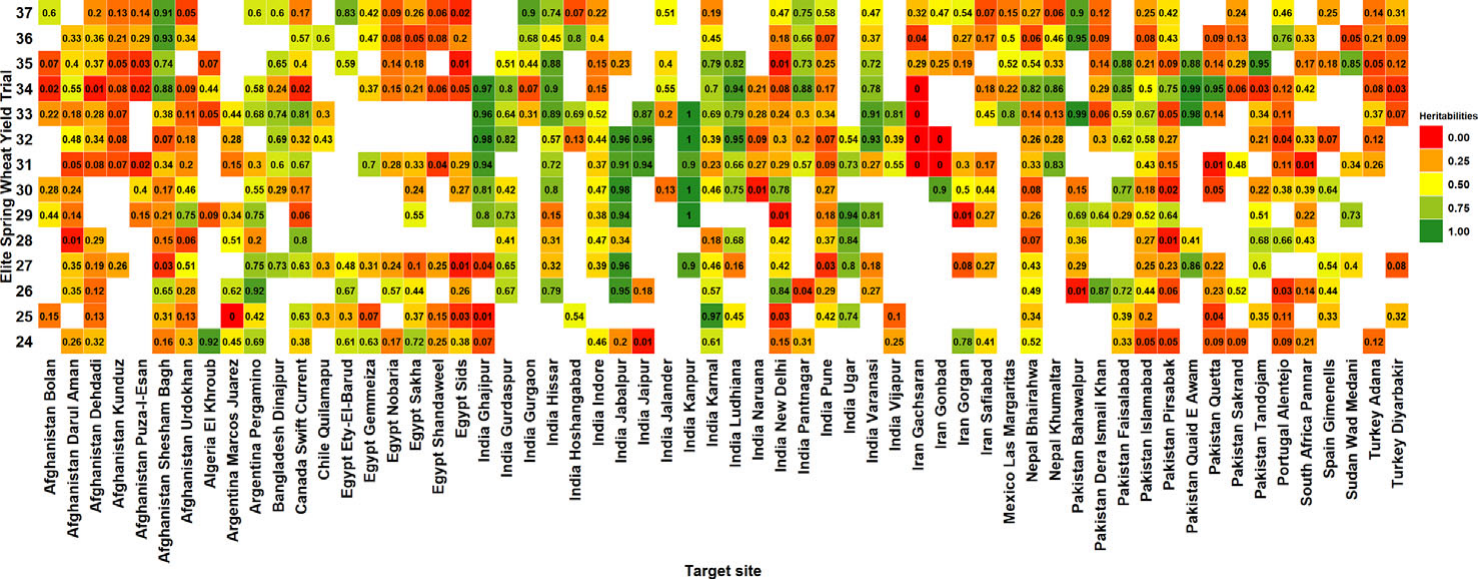
Narrow-sense heritabilities of grain yield in the 24^th^ to the 37^th^ Elite Spring Wheat Yield Trials (ESWYTs) evaluated in 60 target sites indicated by the country followed by the site.

Among the 60 ESWYT sites, 16 (26.7%) had mean heritabilities greater than 0.5, 24 (40%) had mean heritabilities between 0.3 and 0.49 and 20 (33.3%) had mean heritabilities less than 0.3. In addition, we observed high variabilities in the site heritabilities across years and considering all the 519 site-years, 251 site-years (48.4%) had heritabilities less than 0.3, 141 site-years (27.2%) had heritabilities between 0.31 and 0.6, 107 site-years (20.6%) had heritabilities between 0.61 and 0.95, and 20 site-years (3.9%) had unrealistically high heritabilities between 0.95 and 1 (**Figure 2**).

### Genetic Correlations Between the Selection and Target Environments

**Figure 3:**
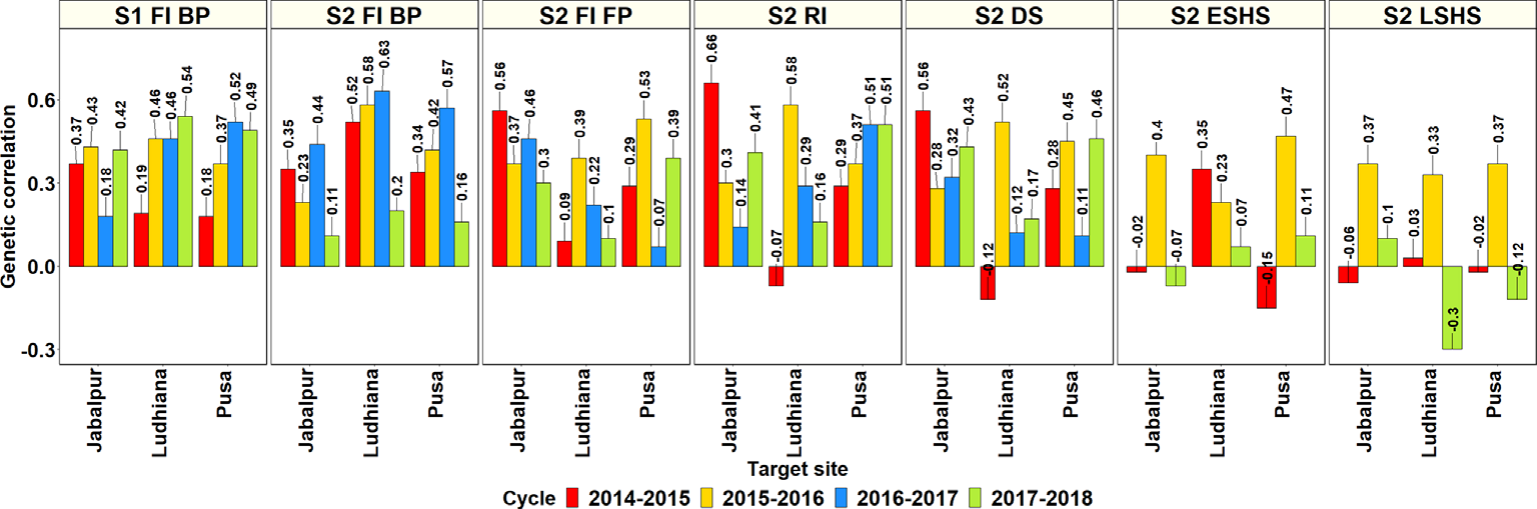
Genetic correlations between the environments in the Stages 1 and 2 of yield testing in Obregon and the three sites in India where the South Asia Bread Wheat Genomic Prediction Yield Trials were evaluated in four cycles. S1 FI BP, Stage 1 full irrigation bed planting; S2 FI BP, Stage 2 full irrigation bed planting; S2 FI FP, Stage 2 full irrigation flat planting; S2 RI, Stage 2 reduced irrigation; S2 DS, Stage 2 drought stress; S2 ESHS, Stage 2 early-sown heat stress; and S2 LSHS, Stage 2 late-sown heat stress.

The GCs between the Stage 1 and Stage 2 SEs in Obregon and the SABWGPYT sites (**Figure 3**, **Supplementary Table 5**) indicated that among the 95 SE-SABWGPYT site combinations, 77 (81%) combinations had a significant GC at a p-value threshold of 0.001. While the Stage 1 irrigated BP environment had significant GCs with all the SABWGPYT sites in all the years, the Stage 2 late heat, Stage 2 early heat, Stage 2 drought, Stage 2 irrigated FP, Stage 2 reduced irrigation, and Stage 2 irrigated BP environments had insignificant GCs in 5, 4, 3, 3, 2, and 1 site-year combinations, respectively. For GY evaluated in Bangladesh Jamalpur, the highest GC (0.41) was with the Stage 2 reduced irrigation environment. For GY evaluated in India Jabalpur, the highest GCs with the SEs ranged between 0.43 and 0.66 and were with the Stage 2 reduced irrigation, Stage 2 irrigated BP, Stage 2 irrigated FP, and Stage 2 drought environments in the four cycles, respectively. For GY evaluated in India Ludhiana, the highest GCs with the SEs ranged between 0.52 and 0.63 and were with the Stage 2 irrigated BP environment in the first three cycles and the Stage 1 irrigated BP environment in the fourth cycle. For GY evaluated in India Pusa, the highest GCs with the SEs ranged between 0.34 and 0.57 and were with the Stage 2 irrigated BP, Stage 2 irrigated FP, Stage 2 irrigated BP, and Stage 2 reduced irrigation environments, respectively. For GY evaluated in Pakistan Faisalabad, the highest GCs in the two cycles were 0.59 and 0.55 and were with the Stage 2 drought and Stage 2 irrigated FP environments. Across all the SABWGPYT sites, the SEs that had high mean GCs were Stage 1 irrigated BP (0.39 ± 0.14), Stage 2 irrigated BP (0.38 ± 0.18), Stage 2 reduced irrigation (0.36 ± 0.18), Stage 2 irrigated FP (0.34 ± 0.17), and Stage 2 drought (0.31 ± 0.2), while the Stage 2 early heat (0.19 ± 0.23) and Stage 2 late heat (0.1 ± 0.23) SEs had low GCs.

In the 36^th^ ESWYT sites, the highest GCs with an Obregon SE were significant in 46 out of the 69 sites (66.7%) at a p-value threshold of 0.05 (the threshold was relaxed because of the small sample size). The highest GCs of these 46 sites with an Obregon SE ranged between 0.3 and 0.4 in 22 sites, between 0.41 and 0.5 in 12 sites and between 0.51 and 0.67 in 12 sites. The 36^th^ ESWYT sites that had the highest GCs with the Obregon SEs included Canada Swift Current (0.67), Algeria Setif (0.66), Turkey Adana (0.65), Egypt Sids (0.62), India Wellington (0.62), and Ethiopia Adet (0.58). The SEs that had the highest significant GCs with the 46 target sites included the Stage 2 irrigated FP (11 sites), Stage 2 early heat (8 sites), Stage 3 irrigated BP (6 sites), Stage 2 drought (5 sites), Stage 2 reduced irrigation (4 sites), Stage 2 irrigated BP (3 sites), Stage 3 drought (3 sites), Stage 3 late heat (3 sites), Stage 1 irrigated BP (2 sites), and the Stage 2 late heat (1 site) environments.

In the 37^th^ ESWYT sites, the highest GCs with an Obregon SE were significant in 45 out of the 71 sites (63.4%) at a p-value threshold of 0.05. The highest GCs of these 45 sites with an Obregon SE ranged between 0.3 and 0.4 in 24 sites, between 0.41 and 0.5 in 16 sites, and between 0.51 and 0.67 in 5 sites. The 37^th^ ESWYT sites that had the highest GCs with the Obregon SEs included Canada Swift Current (0.66), Afghanistan Dehdadi (0.56), Argentina Pergamino (0.56), India New Delhi (0.56) and India Jalander (0.52). The SEs that had the highest significant GCs with the target sites included the Stage 2 early heat (9 sites), Stage 1 irrigated BP (6 sites), Stage 2 irrigated BP (6 sites), Stage 2 reduced irrigation (6 sites), Stage 2 drought (5 sites), Stage 3 late heat (5 sites), Stage 3 irrigated BP (4 sites), Stage 2 irrigated FP (2 sites) and Stage 2 late heat (2 sites) environments.

**Figure 4:**
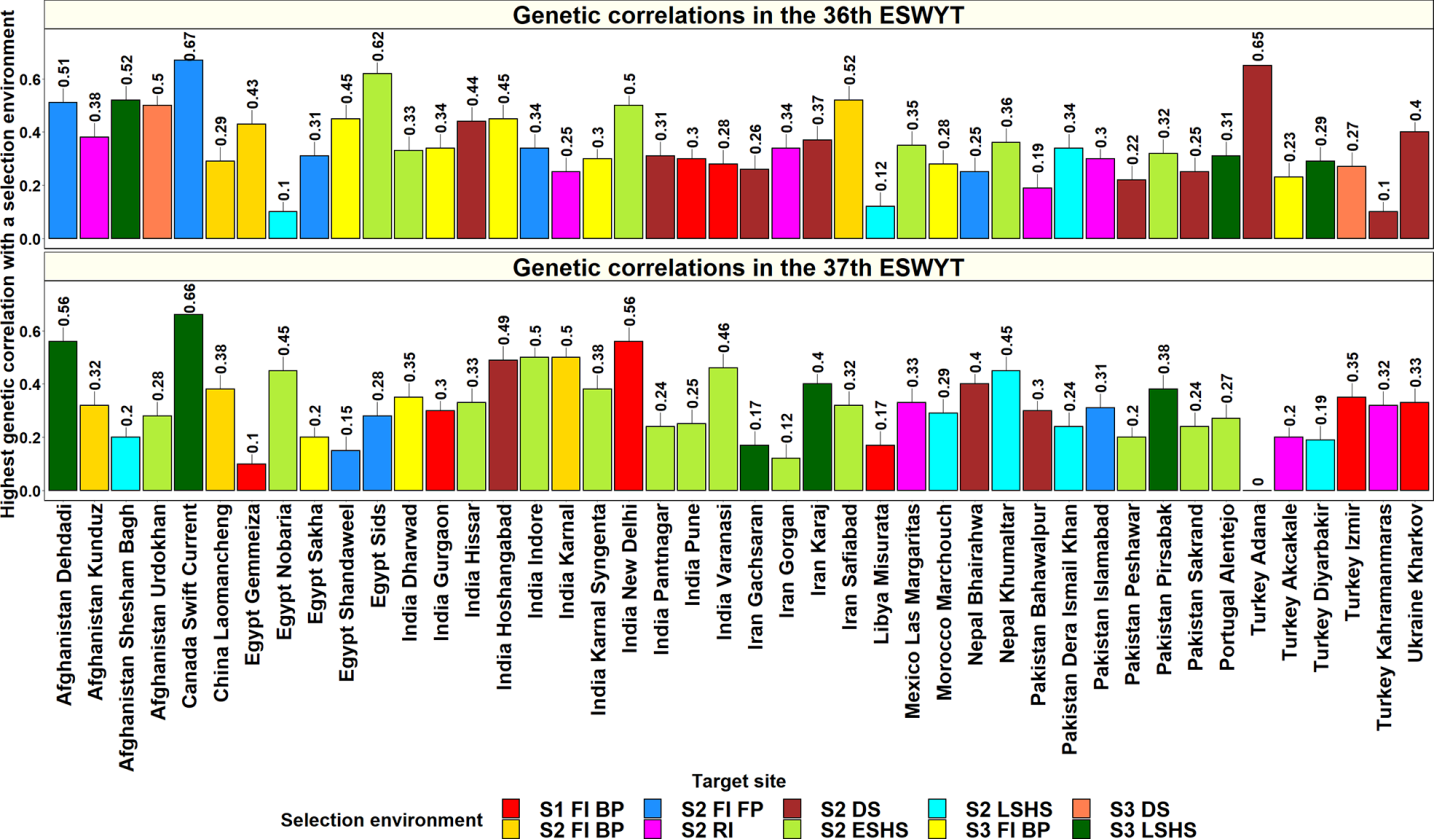
Highest genetic correlations between a selection environment in the Stages 1, 2, and 3 of yield testing in Obregon and the 44 target sites in the Elite Spring Wheat Yield Trials (ESWYTs) where both the 36^th^ and the 37^th^ ESWYT were evaluated. S1 FI BP, Stage 1 full irrigation bed planting; S2 FI BP, Stage 2 full irrigation bed planting; S2 FI FP, Stage 2 full irrigation flat planting; S2 RI, Stage 2 reduced irrigation; S2 DS, Stage 2 drought stress; S2 ESHS, Stage 2 early-sown heat stress; S2 LSHS, Stage 2 late-sown heat stress; S3 FI BP, Stage 3 full irrigation bed planting; S3 DS, Stage 3 drought stress; and S3 LSHS, Stage 3 late-sown heat stress.

Considering the 44 sites where both the 36^th^ and 37^th^ ESWYTs were evaluated, the mean of the highest GCs of these sites with an Obregon SE ranged between 0.15 and 0.3 in 18 sites, between 0.31 and 0.4 in 18 sites, and between 0.41 and 0.67 in 8 sites (**Figure 4**). The sites that had the highest mean GCs with the Obregon SEs and low standard deviations across the two ESWYTs included Canada Swift Current (0.67 ± 0.003), Afghanistan Dehdadi (0.54 ± 0.02), India New Delhi (0.53 ± 0.02), India Hoshangabad (0.47 ± 0.01), India Indore (0.42 ± 0.06), Iran Safiabad (0.42 ± 0.07). and Nepal Khumaltar (0.41 ± 0.03). However, only two among the 44 sites had the highest GCs with the same SE in both the years including China Laomancheng with the Stage 2 irrigated BP environment and Pakistan Dera Ismail Khan with the Stage 2 late heat environment. Overall, the SEs having the highest GCs with the 44 sites in both the years included the Stage 2 early heat (20 sites) and Stage 2 drought (12 sites) environments. We also observed that the Stage 1 irrigated BP, Stage 2 irrigated BP, Stage 3 irrigated BP, Stage 2 reduced irrigation, Stage 2 late heat, and Stage 3 late heat environments had the highest GCs in eight sites each, while the Stage 2 irrigated BP environment had the highest GC in six sites.

**Figure 5:**
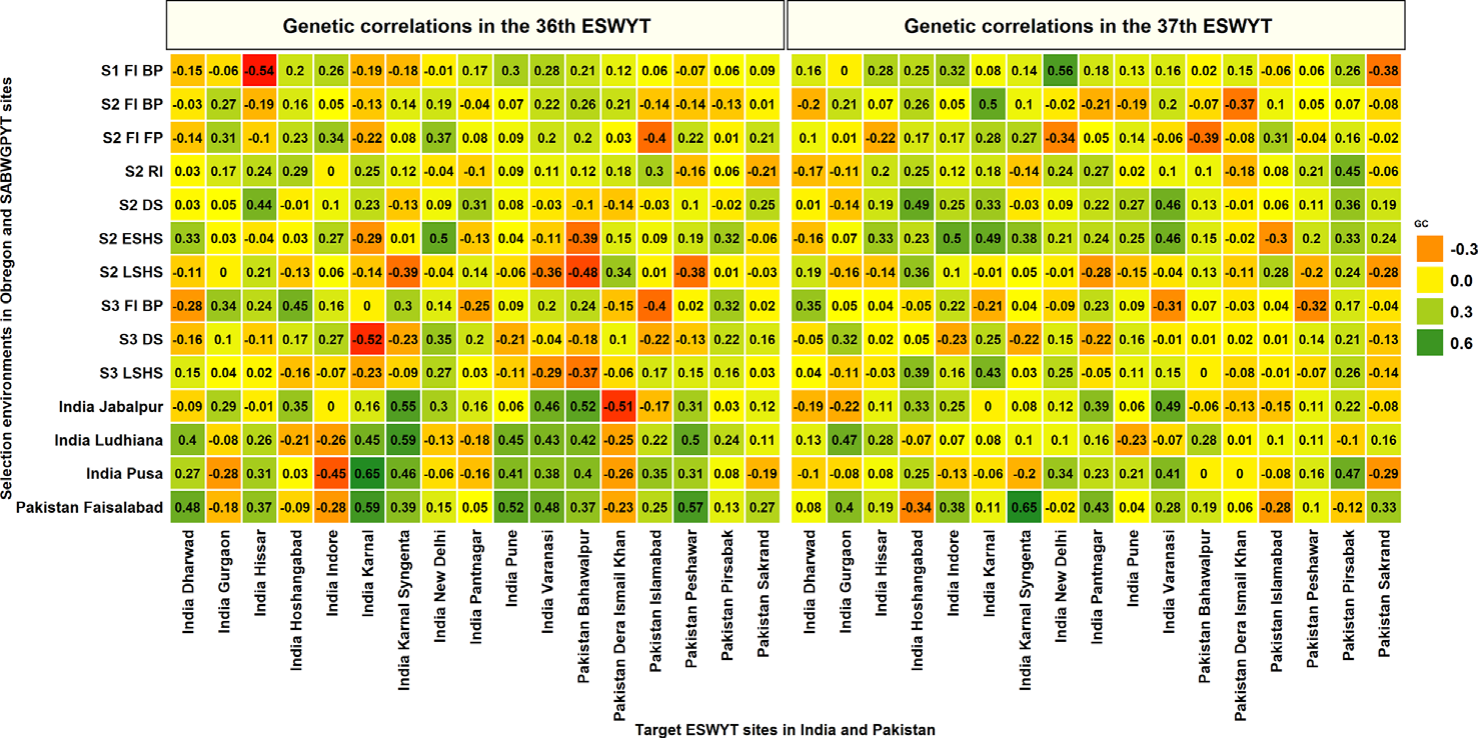
Genetic correlations between grain yield evaluated in the selection environments of Obregon and the South Asia Bread Wheat Genomic Prediction Yield Trial sites in India and Pakistan with the grain yield evaluated in Elite Spring Wheat Yield Trial (ESWYT) sites in India and Pakistan in the 36^th^ and 37^th^ ESWYT. S1 FI BP, Stage 1 full irrigation bed planting; S2 FI BP, Stage 2 full irrigation bed planting; S2 FI FP, Stage 2 full irrigation flat planting; S2 RI, Stage 2 reduced irrigation; S2 DS, Stage 2 drought stress; S2 ESHS, Stage 2 early-sown heat stress; S2 LSHS, Stage 2 late-sown heat stress; S3 FI BP, Stage 3 full irrigation bed planting; S3 DS, Stage 3 drought stress; and S3 LSHS, Stage 3 late-sown heat stress.

To understand the selection abilities of the SABWGPYT sites in India and Pakistan and the Obregon SEs with the target sites in India and Pakistan, we obtained their GCs with 11 ESWYT sites in India and 6 ESWYT sites in Pakistan that were evaluated in both the 36^th^ and the 37^th^ ESWYTs (**Figure 5**). In the 36^th^ ESWYT, the highest GCs of eight of the 17 target sites were with the SEs in Obregon that included India Gurgaon, India Hissar, India Hoshangabad, India Indore, India New Delhi, India Pantnagar, Pakistan Dera Ismail Khan, and Pakistan Pirsabak. In the 37^th^ ESWYT, the highest GCs of 10 target sites were with the SEs in Obregon that included India Dharwad, India Hissar, India Hoshangabad, India Indore, India Karnal, India New Delhi, India Pune, Pakistan Dera Ismail Khan, Pakistan Islamabad, Pakistan Peshawar. The four sites where the SABWGPYT sites had a higher GC compared to the Obregon SEs in both the ESWYTs included India Karnal Syngenta (0.59 with India Ludhiana in the 36^th^ ESWYT and 0.65 with Pakistan Faisalabad in the 37^th^ ESWYT), India Varanasi (0.46 and 0.49 with India Jabalpur in the 36^th^ and 37^th^ ESWYT respectively), Pakistan Bahawalpur (0.52 with India Jabalpur in the 36^th^ ESWYT and 0.28 with India Ludhiana in the 37^th^ ESWYT), and Pakistan Sakrand (0.27 and 0.33 with Pakistan Faisalabad in the 36^th^ and 37^th^ ESWYTs, respectively).

### Genetic Correlations Between the Target Sites and Clustering of Target Sites

The GCs between the target sites in the SABWGPYTs, 36^th^ and 37^th^ ESWYTs were analyzed to identify consistently correlated sites (**Supplementary Tables 7** and **8**). In the SABWGPYT sites, high mean GCs were observed between India Pusa and Bangladesh Jamalpur (0.75), Pakistan Faisalabad and India Pusa (0.68 ± 0.14), Pakistan Faisalabad and India Ludhiana (0.59 ± 0.24), Pakistan Faisalabad and Bangladesh Jamalpur (0.57), and India Pusa and India Ludhiana (0.54 ± 0.16). However, the mean GCs of India Jabalpur with Pakistan Faisalabad (0.44 ± 0.04), India Pusa (0.42 ± 0.18), Bangladesh Jamalpur (0.35), and India Ludhiana (0.22 ± 0.05) were moderate to low.

**Figure 6:**
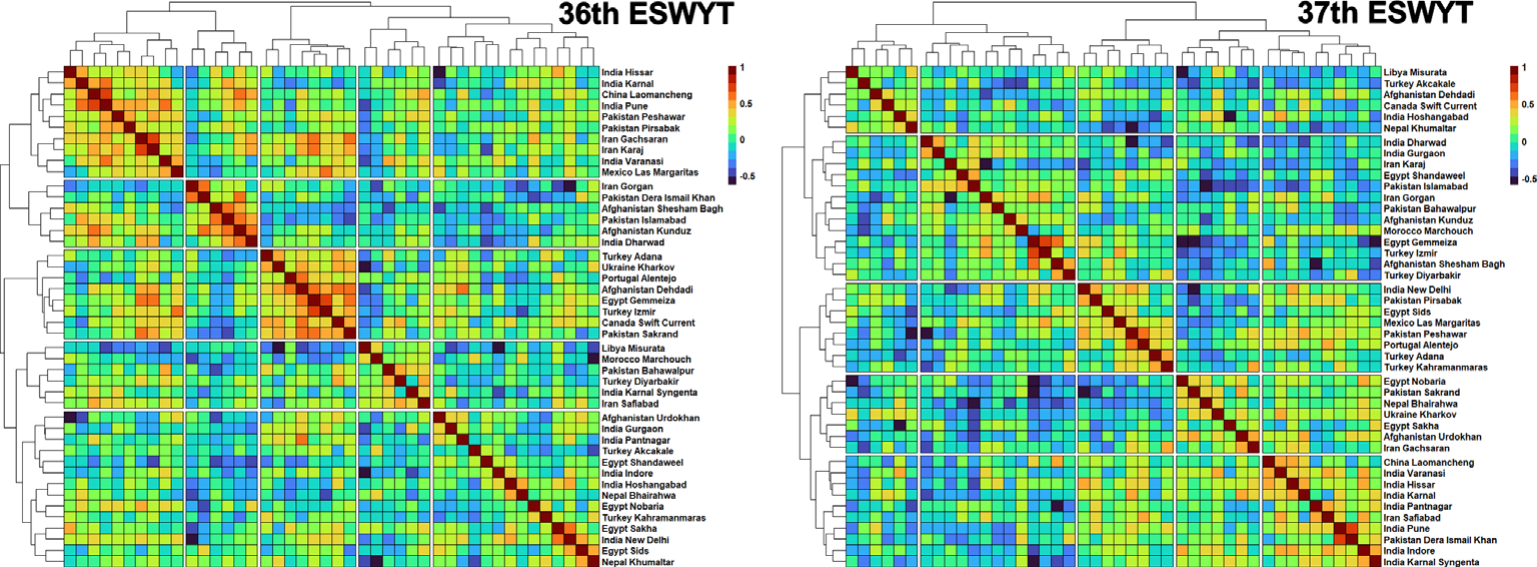
Clustering of the Elite Spring Wheat Yield Trials (ESWYT) sites based on the genetic correlations of grain yield evaluated in those sites with lines from the 36^th^ and the 37^th^ ESWYT.

In the two ESWYTs, the highest mean GCs and low standard deviations across the two ESYWTs were observed between the following target site pairs: Turkey Izmir and Egypt Gemmeiza (0.67 ± 0.01), India Karnal and India Hissar (0.52 ± 0.005), Turkey Izmir and Mexico Las Margaritas (0.52 ± 0.08), Canada Swift Current and Afghanistan Dehdadi (0.48 ± 0.09), Turkey Adana and Turkey Kahramanmaras (0.44 ± 0.09), China Laomancheng and India Karnal (0.44 ± 0.09), Afghanistan Dehdadi and Ukraine Kharkov (0.43 ± 0.1), India Dharwad and Pakistan Islamabad (0.43 ± 0.08), Pakistan Pirsabak and India Pune (0.42 ± 0.07), China Laomancheng and India Varanasi (0.42 ± 0.05), Pakistan Sakrand and Ukraine Kharkov (0.41 ± 0.04), Pakistan Peshawar and India Pune (0.41 ± 0.02), Mexico Las Margaritas and India New Delhi (0.4 ± 0.03), and India Varanasi and India Indore (0.4 ± 0.06). When these sites in the 36^th^ and 37^th^ ESWYTs were clustered based on their GCs, the following pairs clustered together in both the ESWYTs: Turkey Izmir and Egypt Gemmeiza, India Karnal and India Hissar, Canada Swift Current and Afghanistan Dehdadi, China Laomancheng and India Karnal, India Dharwad and Pakistan Islamabad, and Pakistan Sakrand and Ukraine Kharkov (**Figure 6**).

### Ratios of Response to Selection

**Figure 7:**
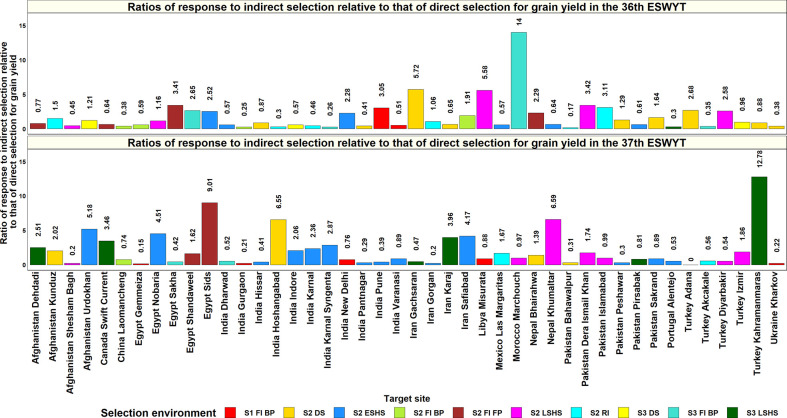
Ratios of response to indirect selection in the selection-environments of Obregon relative to that of direct selection in 44 target-environments, where the 36^th^ and 37^th^ Elite Spring Wheat Yield Trials (ESWYTs) were evaluated. S1 FI BP, Stage 1 full irrigation bed planting; S2 FI BP, Stage 2 full irrigation bed planting; S2 FI FP, Stage 2 full irrigation flat planting; S2 RI, Stage 2 reduced irrigation; S2 DS, Stage 2 drought stress; S2 ESHS, Stage 2 early-sown heat stress; S2 LSHS, Stage 2 late-sown heat stress; S3 FI BP, Stage 3 full irrigation bed planting; S3 DS, Stage 3 drought stress; and S3 LSHS, Stage 3 late-sown heat stress.

The ratios of response indirect selection in the SEs of Obregon relative to that of direct selection in the TPEs were calculated for the SABWGPYT and ESWYT sites (**Supplementary Table 9**, **Figure 7**). For the SABWGPYT sites, the highest ratios of response to indirect selection in Obregon ranged between 0.52 and 1.14 across all the site-years, with a mean of 0.80 ± 0.21. Considering the individual sites, the highest ratios of response to indirect selection in the different years ranged as follows: Bangladesh Jamalpur: 0.98, India Jabalpur: 0.53 to 1.14, India Ludhiana: 0.64 to 0.86, India Pusa: 0.52 to 1.1, and Pakistan Faisalabad: 0.94 to 0.98. The Obregon SEs that had the highest ratios of indirect selection response for the SABWGPYTs included Stage 2 drought environment (4 site-years), Stage 2 irrigated BP environment (3 site-years), and Stage 2 reduced irrigation environment (3 site-years).

In the ESWYT sites, the mean of the highest ratios of response to indirect selection in the SEs of Obregon across two cycles was 2.6 ± 5.4. In the 36^th^ ESWYT, the ratios of response to indirect selection were greater than one for 34 sites (49.3%) and greater than 0.5 for 50 sites (72.5%). While the highest ratios of response for indirect response (greater than five) were observed for Ethiopia Adet, Morocco Marchouch, Zambia Lusaka, Iran Gachsaran, Libya Misurata, and India Wellington, sites like Angola Chianga, Iraq Bakrajo, Pakistan Bahawalpur, and Spain Castilla Y Leon had the lowest ratios. The Obregon SEs that had the highest ratios of indirect selection response for the 36^th^ ESWYT sites included Stage 2 early heat (11sites), Stage 2 drought (10 sites), Stage 3 drought (10 sites), and Stage 2 irrigated FP (8 sites). In the 37^th^ ESWYT, the ratios of response to indirect selection were greater than one for 31 sites (43.7%) and greater than 0.5 for 52 sites (73.2%). Sites like Nigeria Birnin Kebbi, Afghanistan Takhar, India Nagpur, Turkey Kahramanmaras, Egypt Sids, Nepal Khumaltar, India Hoshangabad, and Afghanistan Urdokhan had the highest ratios for indirect selection, while the lowest ratios were observed for sites like Turkey Adana, Egypt Gemmeiza, Afghanistan Shesham Bagh, and Iran Gorgan. The Obregon SEs that had the highest ratios of indirect selection response for the 37^th^ ESWYT sites included the Stage 2 early heat (20 sites), Stage 2 drought (11 sites), Stage 2 late heat (10 sites), and Stage 3 late heat (9 sites) environments. The means of the highest ratios of response to indirect selection in sites with two years of evaluations indicated that 66% of the 44 sites had mean response ratios greater than one and 82% of the sites had ratios greater than 0.5. Overall, across both the ESWYTs, the Obregon SEs that had the highest ratios of indirect response to selection were Stage 2 early heat (20 site-years), Stage 2 drought (13 site-years), Stage 2 late heat (12 site-years), and Stage 2 reduced irrigation (8 sites) environments.

### Genomic Prediction for Grain Yield in the Target Sites Using Their Yields in the Selection-Environments of Obregon

**Figure 8:**
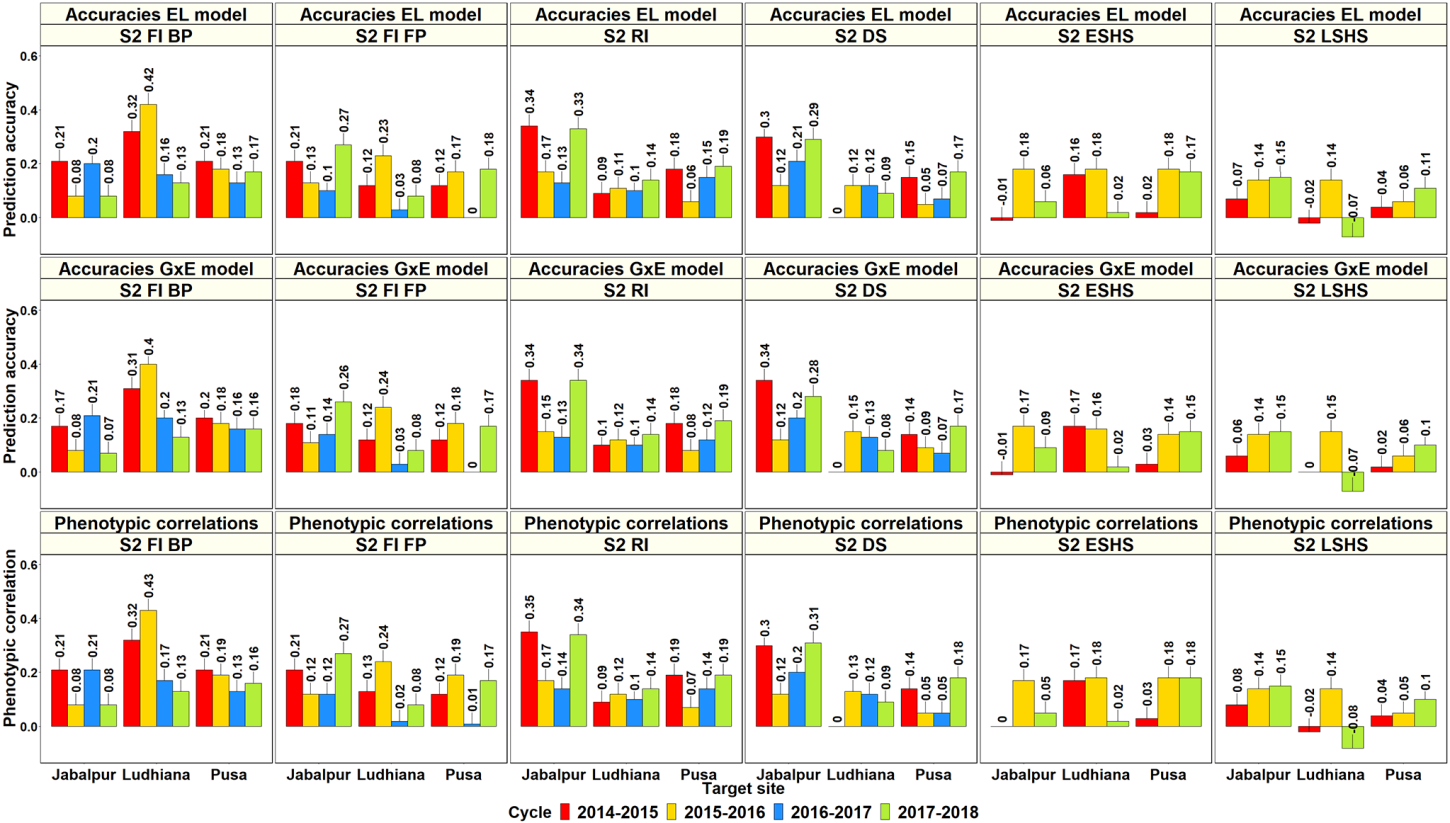
Prediction accuracies for grain yield in the South Asia Bread Wheat Genomic Prediction Yield Trial target sites predicted from the yield of the same lines (487 to 515 lines) in the selection-environments of Obregon using the baseline model with environment and line effects and the model with environment, genomic, and genotype x interaction (GxE) interaction effects (GxE model), along with the phenotypic correlations between the selection and target-environments.

The ability of GY evaluated in the SEs of Obregon to predict GY in the target SABWGPYTs and ESWYTs were evaluated for 1,424 SE-TPE pairs and compared to the phenotypic correlations between the environments (**Supplementary Table 10**). In the SABWGPYT evaluation sites where the GY of 487 to 515 lines were predicted from their corresponding Obregon yields, the mean of the highest PAs from the Obregon SEs using the GxE model across the five South-Asian sites was 0.24 ± 0.08 and the mean PAs were the highest in India Ludhiana (0.28 ± 0.1), followed by Pakistan Faisalabad (0.28 ± 0.11), India Jabalpur (0.27 ± 0.08), India Pusa (0.19 ± 0.01), and Bangladesh Jamalpur (0.13). The Obregon SEs that had the highest PAs for the SABWGPYT sites included the Stage 2 irrigated BP environment (7 site-years) and the Stage 1 irrigated BP environment (3 site-years) (**Figure 8**). A strong relationship between the PAs from the GxE model and the PAs from the EL model (correlation of 0.98) and the phenotypic correlations (correlation of 0.98) was observed and there were no significant differences between the PAs from the GxE model and the EL model (p-value of 0.47) and between the PAs from the GxE model and the phenotypic correlations (p-value of 0.73).

To further understand the PAs from the GxE and EL models, we partitioned the GY variance between the SABWGPYT sites and the Obregon SE that had the highest predictability for each site into the genetic, environment (including year and site), genotype x environment, and error variance components and obtained the ratios of the variance components relative to the genetic variance (**Table 1**). We observed that the environmental variance between the Obregon SEs with the highest predictabilities and the SABWGPYT sites were high and as follows: Bangladesh Jamalpur (23.8 times the genetic variance), Pakistan Faisalabad (7.3 to 19.2 times the genetic variance), India Jabalpur (3.6 to 37.1 times the genetic variance), and India Pusa (5.6 to 9.4 times the genetic variance). However, the environmental variance between the Obregon SE with the highest predictability and India Ludhiana was low (1.1 to 3.3 times the genetic variance). We also observed that the GxE variance was 0.4 to 1.8 times the genetic variance, and the error variance was 0.8 to 3.8 times the genetic variance across the sites. The mean narrow-sense heritabilities between the SEs with the highest predictabilities and the SABWGPYT sites were high in India Ludhiana (0.47 ± 0.12), followed by India Pusa (0.22 ± 0.05), India Jabalpur (0.2 ± 0.16), Pakistan Faisalabad (0.15 ± 0.08), and Bangladesh Jamalpur (0.08).

**Table 1:** Marker-based estimates of genetic (σ^2^_g_), environment (σ^2^_e_), genotype x environment (σ^2^_gxe_) and error (σ^2^_ϵ_) variance components, variance ratios, and narrow-sense heritabilities across the target South Asia Bread Wheat Genomic Prediction Yield Trial sites and the Obregon selection environment that had the highest prediction accuracy for the target site.

Variance components, variance ratios, and heritabilities	Cycle	Bangladesh Jamalpur	India Jabalpur	India Ludhiana	India Pusa	Pakistan Faisalabad
σ^2^_g_	2014–2015		0.08	0.10	0.10	
σ^2^_e_			1.74	0.33	0.90	
σ^2^_gxe_			0.06	0.07	0.09	
σ^2^_ϵ_			0.11	0.11	0.16	
σ^2^_g_:σ^2^_e_:σ^2^_gxe_:σ^2^_ϵ_			1:21.8:0.8:1.3	1:3.3:0.7:1.2	1:9.4:0.9:1.7	
*h^2^*			0.08	0.38	0.18	
σ^2^_g_	2015–2016	0.05	0.09	0.13	0.08	0.03
σ^2^_e_		1.16	0.32	0.15	0.46	0.18
σ^2^_gxe_		0.06	0.10	0.05	0.09	0.04
σ^2^_ϵ_		0.09	0.18	0.10	0.14	0.06
σ^2^_g_:σ^2^_e_:σ^2^_gxe_:σ^2^_ϵ_		1:23.8:1.3:2	1:3.6:1.1:2	1:1.1:0.4:0.8	1:5.8:1.1:1.8	1:7.3:1.5:2.5
*h^2^*		0.08	0.36	0.64	0.26	0.21
σ^2^_g_	2016–2017		0.04	0.05	0.05	0.08
σ^2^_e_			0.18	0.14	0.26	1.45
σ^2^_gxe_			0.05	0.09	0.07	0.08
σ^2^_ϵ_			0.17	0.11	0.12	0.13
σ^2^_g_:σ^2^_e_:σ^2^_gxe_:σ^2^_ϵ_			1:4.1:1.2:3.8	1:2.8:1.8:2.1	1:5.6:1.5:2.6	1:19.2:1:1.8
*h^2^*			0.33	0.42	0.26	0.09
σ^2^_g_	2017–2018		0.11	0.05	0.05	
σ^2^_e_			3.90	0.13	0.41	
σ^2^_gxe_			0.10	0.07	0.05	
σ^2^_ϵ_			0.12	0.11	0.10	
σ^2^_g_:σ^2^_e_:σ^2^_gxe_:σ^2^_ϵ_			1:37.1:0.9:1.2	1:2.6:1.4:2.2	1:8.8:1.1:2.1	
*h^2^*			0.05	0.44	0.19	

**Figure 9:**
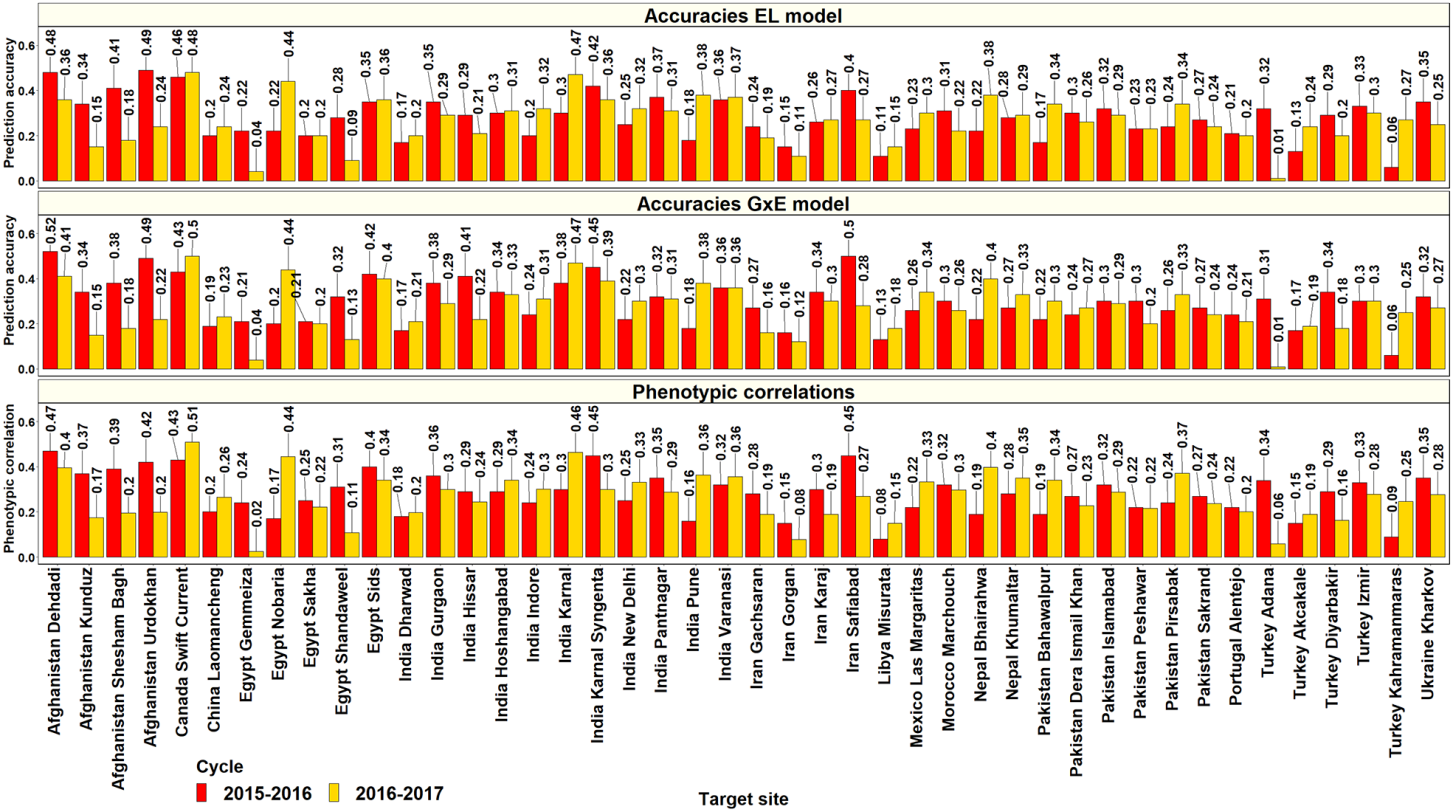
Prediction accuracies for grain yield in the Elite Spring Wheat Yield Trial target sites predicted from the yield of the same lines (42 lines in the 2015–2016 cycle and 43 lines from the 2016–2017 cycle) in the selection-environments of Obregon using the baseline model with environment and line effects and the model with environment, genomic, and genotype x interaction (GxE) interaction effects (GxE model), along with the phenotypic correlations between the selection and target-environments.

In the 36^th^ ESWYT, where the yields of 42 lines were predicted from their corresponding yields in the Obregon SEs, the mean of the highest PAs from the GxE model was 0.29 ± 0.11 (ranged between 0.06 and 0.56) and the best predicted sites were Venezuela Los Bagres (0.56), Afghanistan Dehdadi (0.52), Pakistan Quetta (0.51), Iran Safiabad (0.5), and Afghanistan Urdokhan (0.49). The Obregon SEs that had the highest PAs for the sites in the 36^th^ ESWYT were: Stage 2 irrigated FP (19 sites), Stage 2 drought (8 sites), Stage 3 irrigated BP (8 sites), and Stage 2 early heat (7 sites). In the 37^th^ ESWYT, where the yields of 43 lines were predicted, the mean of the highest PAs from the GxE model was 0.29 ± 0.1 (ranged between 0.01 and 0.63), and the sites that had the highest PAs included Argentina Pergamino (0.63), Canada Swift Current (0.5), India Karnal (0.47), and Egypt Nobaria (0.44). The Obregon SEs that had the highest PAs for the 37^th^ ESWYT sites included Stage 2 early heat (16 sites), Stage 2 reduced irrigation (10 sites), Stage 1 irrigated BP (9 sites), Stage 2 drought (7 sites), and Stage 2 late heat (7 sites). Considering only the target sites where the ESWYTs were evaluated in both the years, the mean of the highest PAs from the Obregon SEs using the GxE model was 0.28 ± 0.08 (**Figure 9**). On comparing the PAs from the GxE model with the baseline EL model across both the ESWYT sites, we observed a high correlation (0.95) and a negligible mean increase using the GxE model (0.01 ± 0.03), despite significant differences between the pairwise accuracies (p-value for the test of significance of differences between them was 4.05e-5). We also observed a high correlation between the PAs from the GxE model and the phenotypic correlations between the environments (0.94) and insignificant differences between them (p-value for the test of significance of differences between them was 0.02).

## Discussion

We have performed a large retrospective quantitative genetics study that provides excellent insights into the effectiveness of the current GY testing strategies adopted by the CIMMYT wheat breeding program and the screening ability of CIMMYT’s key GY testing site. A remarkable increase in the mean GY (ranging between 27.3% and 40.5%) from the base GY in 2014–2015 was observed across four years at the SABWGPYT sites, clearly indicating continuous GY improvement at the target South Asia sites by indirect selection in the SEs of Obregon. While we also observed a 24% increase in the mean GY across 14 years in the ESYWT sites, the high non-linear trends across years in some ESWYT sites stemmed from the different biotic and abiotic stresses prevalent in the sites in different years, changing environments (Braun et al., 1992), variable trial management, and agricultural practices across years etc. Nonetheless, the 445.5% increase in mean GY at an ESWYT site over 12 years and the outstanding performance of the highest yielding ESYWT lines over the local check varieties in 94.7% of the 152 site-year combinations, affirm the high GY performance of the ESWYT lines as also observed in previous studies (Rajaram and Skovmand, 1977; Trethowan et al., 2002; Singh et al., 2007; Crespo-Herrera et al., 2017) and indicate progress made from indirect selection for GY in Obregon.

In the SEs of Obregon, we observed consistent narrow-sense heritabilities across years ranging from 0.5 to 0.94 and the mean heritability in the SEs was 44.2% and 92.3% higher than the mean heritabilities in the SABWGPYT and ESWYT sites, respectively. On the contrary, only a small percentage (26.7%) of the 60 target ESWYT sites had mean heritabilities greater than 0.5, and there were tremendous variations in the heritabilities across years. We also observed significant GCs between a SE in Obregon and all the SABWGPYT sites, 66.7% of the 36^th^ ESWYT sites and 63.4% of the 37^th^ ESWYT sites, clearly demonstrating the efficiency of indirect selection in Obregon. While most SEs had high GCs with several target sites, the observed low GCs between the Stage 2 late heat environment and the target sites was expected, because this SE was not designed to select lines for the optimum environments, but only for environments vulnerable to terminal heat stress.

Our results indicated that except for one SABWGPYT site (India Ludhiana) and two ESWYT sites (China Laomancheng and Pakistan Dera Ismail Khan) that had high GCs with the same SEs in Obregon across years, all the other target sites had high GCs with different SEs in different years. In addition, we also observed non-repeatable GCs between the selection and target environments in different years. For example, the Stage 2 irrigated FP environment that had high GCs with 11 sites in the 36^th^ ESWYT had high GCs with only two sites in the 37^th^ ESWYT. Likewise, the Stage 2 early heat environment which had insignificant GCs with several SABWGPYT sites had the highest GCs with a large number of ESWYT sites. All these observations clearly indicate that the identification of optimal or few SEs that will have high GCs with the target sites in all the years is not feasible and highlights the importance of multiple SEs in breeding for unpredictable changing environments with high year-to-year variations.

We also investigated the relative GCs of the SABWGPYT sites in India and Pakistan and the SEs in Obregon with target sites in India and Pakistan and observed that the SEs in Obregon had the highest GCs with 47% of the sites in the 36^th^ ESWYT and 59% of the sites in the 37^th^ ESWYT. The SABWGPYT sites had the highest GCs in both the ESWYTs with some sites like India Karnal Syngenta, India Varanasi, Pakistan Bahawalpur, and Pakistan Sakrand, which is expected because of the geographical proximities of these locations and the similar wheat growing conditions in some of them. While these results indicate that earlier screening in the SABWGPYT sites provide useful information in selecting lines for some target sites, we also present substantial evidence for the consistent and competitive screening ability of Obregon, which also has the capacity to screen a larger number of lines than the SABWGPYTs.

The GCs between the target sites were also used to understand the similarities between them based on the discrimination of genotypes and to determine the repeatabilities of site clusterings, both of which are important for designing efficient germplasm targeting strategies (DeLacy et al., 1993; Mirzawan et al., 1994; Lillemo et al., 2004). While we observed inconsistent GCs across several ESWYT sites in the two years indicating high year-to-year variabilities and a marked tendency of the sites to change into a different ME due to climate change (Braun et al., 2010), we also observed high and consistent GCs and similar clustering patterns between sites in different MEs like Canada Swift Current (ME6) and Afghanistan Dehdadi (ME12 and ME9), China Laomancheng (ME 6) and India Karnal (ME 1), Afghanistan Dehdadi and Ukraine Kharkov (ME 11), Pakistan Pirsabak (ME4, ME8) and India Pune (ME 5), etc. This provides striking evidence to the wide adaptability of the ESWYT lines to very different geographical regions and MEs (Krull et al., 1968; Braun et al., 1992; Crespo-Herrera et al., 2017), thereby exemplifying the successful GY testing strategies of CIMMYT.

We have reported high ratios of response to indirect selection in the SEs of Obregon with a mean of 0.80 ± 0.21 and 2.6 ± 5.4 in the SABWGPYT and ESWYT sites, respectively. Furthermore, our results indicating that greater than 0.5 ratio of response to indirect selection in Obregon can be achieved for all the SABWGPYT sites and 82% of the ESWYT sites, provide strong evidence to the selection ability of the Obregon SEs. The high ratios of correlated response to indirect selection in Obregon were driven by the moderate to high GCs with the target environments and the high heritabilities in the SEs compared to the TPEs (Falconer, 1960). However, it should also be noted that the ESWYTs used in this study are small populations that were not designed for research purposes (Fox, 1994) and larger populations are necessary to obtain more precise estimates of the response to selection. Nevertheless, these results imply that CIMMYT’s strategy of GY testing in a key site for a range of target environments is appropriate and an ideal strategy considering the cost and resources that will be needed for large-scale GY testing in multiple sites.

We also performed genomic prediction for GY in the target sites using the performance of the lines in the SEs of Obregon and observed moderate mean PAs of 0.24 ± 0.08 and 0.28 ± 0.08 in the SABWGPYT and ESWYT sites, respectively using the GxE model. However, we observed similar PAs using the baseline EL model and no advantage of modeling GxE interactions in this scenario similar to the results reported by Dawson et al. (2013), but contrary to some studies that report a marginal increase using GxE models in other scenarios (Burgueño et al., 2012; Heslot et al., 2013; Jarquín et al., 2017). Moreover, the partitioned phenotypic GY variance components also indicated a high environmental variance (1.1 to 37.1 times the genetic variance) and low GxE variance (0.4 to 1.8 times the genetic variance) in accordance with previous observations (Fowler and De la Roche, 1975; Goodchild and Boyd, 1975), which explains the similarity in PAs using both the models. These are key findings in our study substantiating that environmental variabilities constituted by fluctuating factors like temperature, nutrient, edaphic, rainfall patterns, stresses, and management conditions (Hill, 1975; Bell and Fischer, 1994; Storlie and Charmet, 2013) play a larger role in determining GY compared to GxE interactions. Furthermore, our results also imply that a line’s GY performance in a new environment is predictable only when the effect of the environment is known beforehand, and the effect of GxE interactions do not add much value to the predictabilities. Further studies on evaluating genomic prediction for GY in earlier generations where GY testing is not feasible due to limited seed are needed to understand the application of genomic prediction for GY testing. Overall, this study provides extensive quantitative genetic evidence on the suitability of the Obregon SEs in breeding for GY and provides important insights into the genomic predictabilities of GY in different environments. The high year-to-year fluctuations observed highlight the non-feasibility of breeding for every micro-environment (Rajaram and Skovmand, 1977) and affirm the necessity to breed for GY stability and wide adaptability by multi-environment testing across time and space (Finlay and Wilkinson, 1963; Hurd, 1969; Eskridge, 1990; Kang and Pham, 1991).

## Data Availability Statement

The datasets presented in this study can be found in online repositories. The names of the repository/repositories and accession number(s) can be found in the article/supplementary material.

## Author Contributions

PJ planned the study, performed the analyses, and drafted the manuscript. RS, H-JB, and JH-E supervised the work and designed the experiments. LC-H, UK, TP, AJ, MI, and MR were involved in generating and organizing the phenotyping data. JP and SS were involved in generating the genotyping data. All authors contributed to the article and approved the submitted version.

## Funding

This research was supported by the Delivering Genetic Gain in Wheat (DGGW) project (funded by the Bill and Melinda Gates Foundation and the United Kingdom Department for International Development (DFID) and managed by Cornell University) under the terms of Contract No. OPP1133199 and Feed the Future project through the U.S. Agency for International Development (USAID), under the terms of Contract No. AID-OAA-A-13-00051. The opinions expressed herein are those of the authors and do not necessarily reflect the views of the USAID.

## Conflict of Interest

The authors declare that the research was conducted in the absence of any commercial or financial relationships that could be construed as a potential conflict of interest.
