# Supplementary figures and images for: Retrospective Quantitative Genetic Analysis and Genomic Prediction of Global Wheat Yields
Source: Front Plant Sci. 2020 Aug 27;11:580136. doi: 10.3389/fpls.2020.580136 (PMC7481575; doi:10.3389/fpls.2020.580136)

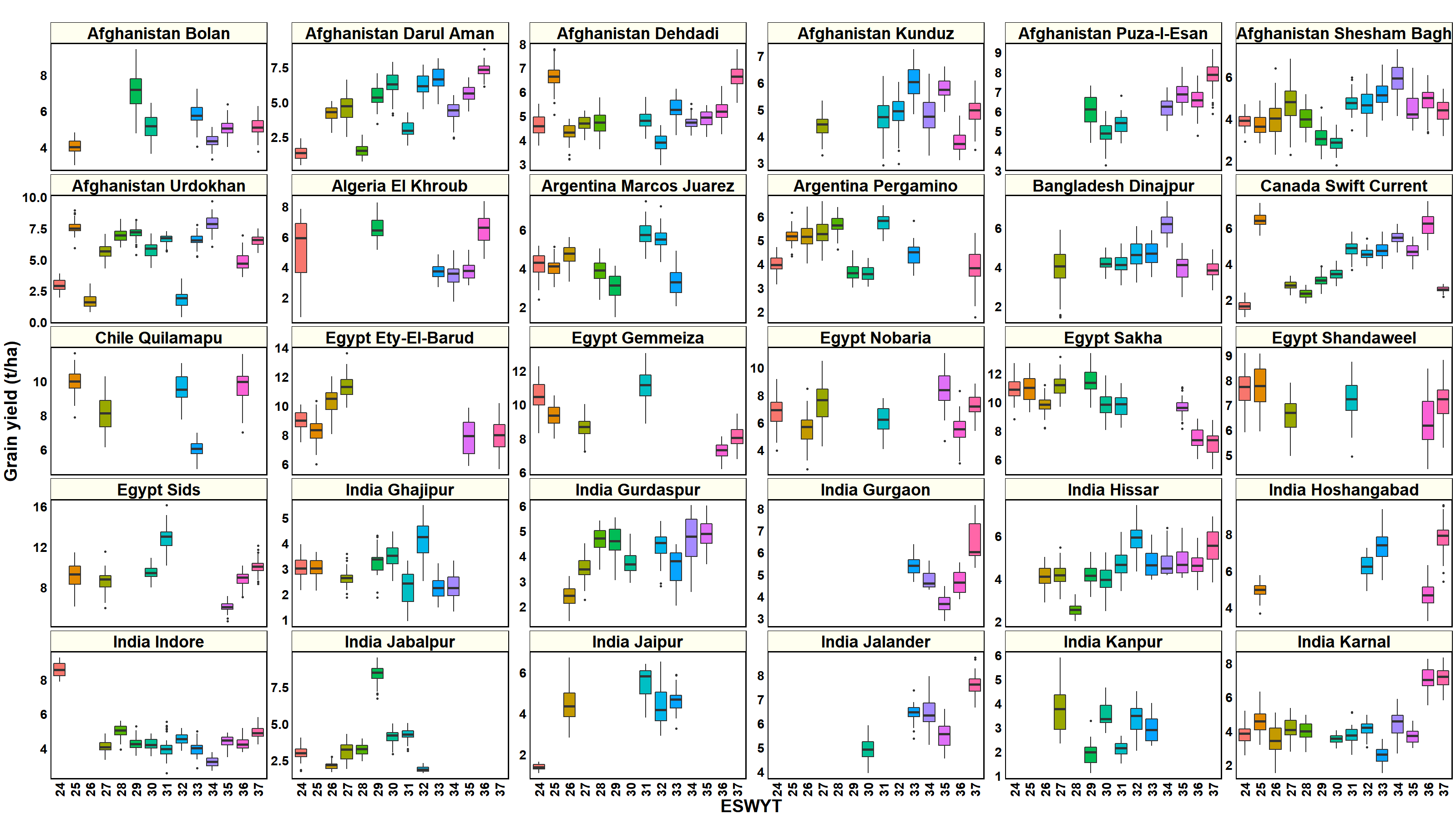

Supplement: Supplementary file 1 [file Image_1.tiff]

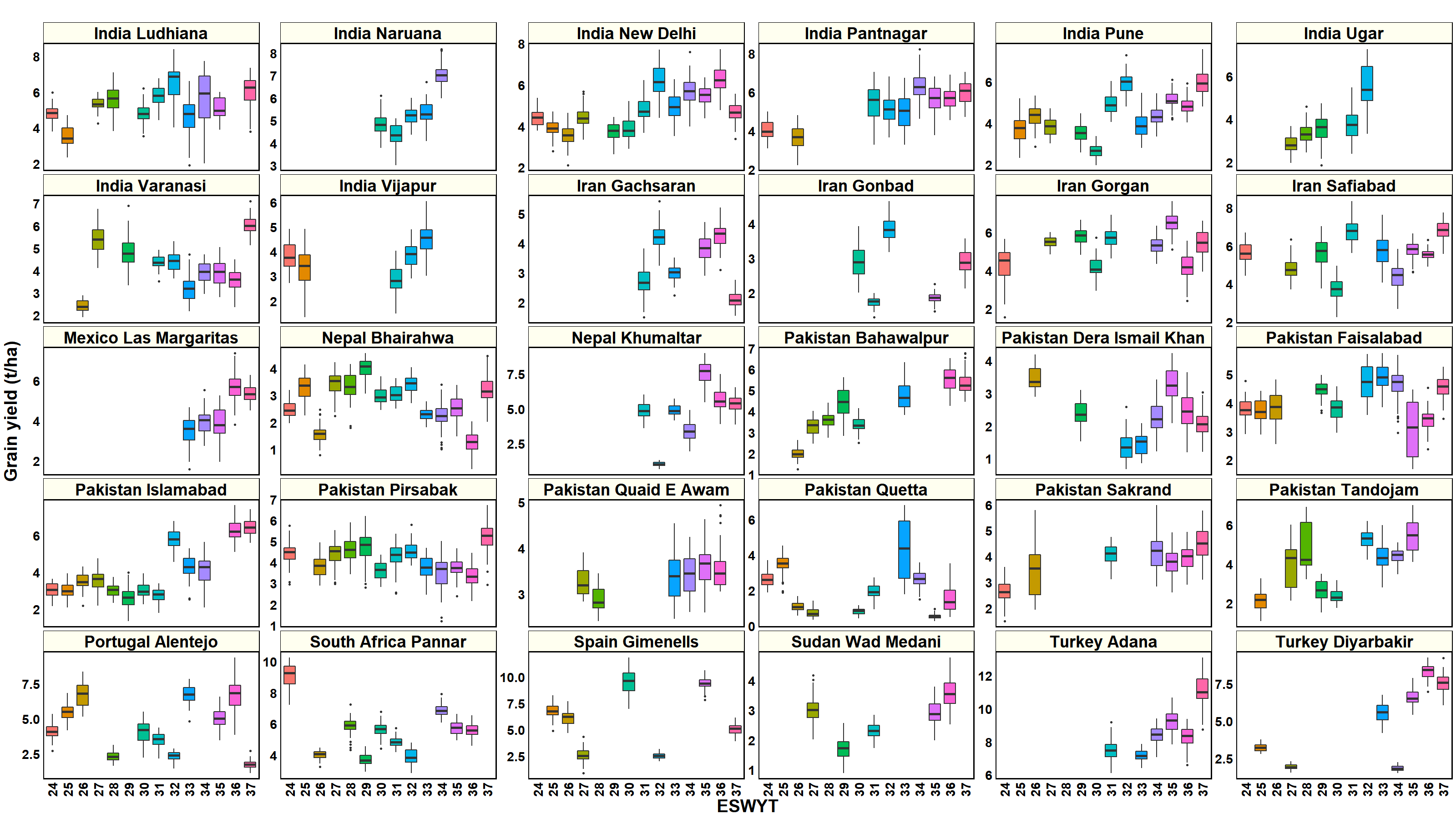

Supplement: Supplementary file 2 [file Image_2.tiff]
